# Suitable indications of eculizumab for patients with refractory generalized myasthenia gravis

**DOI:** 10.1177/1756286420904207

**Published:** 2020-03-18

**Authors:** Munenori Oyama, Kensuke Okada, Masayuki Masuda, Yuko Shimizu, Kazumasa Yokoyama, Akiyuki Uzawa, Naoki Kawaguchi, Ryotaro Ikeguchi, Yasunobu Hoshino, Taku Hatano, Yukiko Ozawa, Jin Nakahara, Hitoshi Aizawa, Kazuo Kitagawa, Nobutaka Hattori, Satoshi Kuwabara, Hiroyuki Murai, Shigeaki Suzuki

**Affiliations:** Department of Neurology, Keio University School of Medicine, Tokyo, Japan; Department of Neurology, Keio University School of Medicine, Tokyo, Japan; Department of Neurology, Tokyo Medical University Hospital, Tokyo, Japan; Department of Neurology, Tokyo Women’s Medical University School of Medicine, Tokyo, Japan; Department of Neurology, Juntendo University School of Medicine, Tokyo, Japan; Department of Neurology, Graduate School of Medicine, Chiba University, Chiba, Japan; Department of Neurology, Neurology Clinic Chiba, Chiba, Japan; Department of Neurology, Tokyo Women’s Medical University School of Medicine, Tokyo, Japan; Department of Neurology, Juntendo University School of Medicine, Tokyo, Japan; Department of Neurology, Juntendo University School of Medicine, Tokyo, Japan; Department of Neurology, Graduate School of Medicine, Chiba University, Chiba, Japan; Department of Neurology, Keio University School of Medicine, Tokyo, Japan; Department of Neurology, Tokyo Medical University Hospital, Tokyo, Japan; Department of Neurology, Tokyo Women’s Medical University School of Medicine, Tokyo, Japan; Department of Neurology, Juntendo University School of Medicine, Tokyo, Japan; Department of Neurology, Graduate School of Medicine, Chiba University, Chiba, Japan; Department of Neurology, School of Medicine, International University of Health and Welfare, 4-3 Kozunomori, Narita, 286-8686, Japan; Department of Neurology, Keio University School of Medicine, 35 Shinanomachi, Shinjuku-ku, Tokyo, 160-8582, Japan

**Keywords:** eculizumab, indication, myasthenia gravis, myasthenic crisis, thymoma

## Abstract

**Background:**

Eculizumab is a humanized monoclonal antibody that targets complement protein C5 and inhibits terminal complement-mediated damage at the neuromuscular junction. Recently, the REGAIN study showed that eculizumab was effective and well tolerated in patients with anti-acetylcholine receptor antibody-positive refractory generalized myasthenia gravis (gMG). However, there is no consensus regarding which kind of patients with gMG are selected to preferentially receive eculizumab.

**Methods:**

Between January and December 2018, we followed 1388 patients with MG at seven hospitals located in Tokyo and Chiba. We evaluated the clinical features of MG and the patients’ quality of life. Clinical status and severity were determined by the recommendations of the Myasthenia Gravis Foundation of America.

**Results:**

Of 1388 patients with MG, 12 (0.9%) patients received eculizumab. A total of 11 patients who were anti-acetylcholine receptor antibody-positive with refractory gMG (M:F = 3:8) completed the 26-week treatment with eculizumab. The disease subtypes represented included five cases of early onset MG, one of late-onset MG, and five of thymoma-associated MG. Overall, seven patients had experienced myasthenic crisis. The mean quantitative MG score ranged from 18.6 at baseline to 9.1 at week 26 (*p* = 0.008). Similarly, the mean MG activities of daily living score ranged from 10.8 at baseline to 4.2 at week 26 (*p* = 0.002). There were marked improvements in all patients’ quality of life status. Overall, seven patients were able to reduce the dose of prednisolone at week 26. All but one patient did not require additional rescue treatment. Overall, one patient with early onset MG could not continue the eculizumab treatment due to nausea and vertigo.

**Conclusion:**

We demonstrate that eculizumab provided remarkable benefits for refractory gMG in practical real-world experience as well as in the REGAIN study. Patients with refractory gMG with myasthenia crisis and thymoma-associated MG are suitable for eculizumab administration.

## Introduction

Generalized myasthenia gravis (gMG) is a chronic autoimmune disorder that is characterized by severe muscle weakness.^1^ In this disease, the complement-mediated pathological membrane changes reduce the efficiency of neurotransmission at the neuromuscular junction, resulting in the characteristic muscle weakness and fatigability. Patients with refractory gMG, representing approximately 10–15% of all patients with MG, do not respond to long-term treatment with corticosteroids or multiple steroid-sparing immunosuppressive therapies (ISTs), or they have intolerable side effects to these therapies or require ongoing treatment with either intravenous immunoglobulin (IVIg) or plasmapheresis.^2^ Patients with refractory gMG might also have frequent exacerbations, which can be life-threatening, require admission to a hospital or intensive care, and cause episodes of respiratory failure that require mechanical ventilation.

Evidence showing that functional blockade of the complement protein C5 protects against severe disease in experimental MG models suggests that complement inhibition might be a potential therapeutic approach for MG.^3,4^ Eculizumab is a monoclonal antibody that specifically binds with high affinity to human terminal complement protein C5, inhibiting enzymatic cleavage to the proteins C5a and C5b and preventing C5a-induced chemotaxis and formation of the C5b-induced membrane attack complex.^5^ Recently, eculizumab was shown to have efficacy and was well tolerated in the 6-month randomized, double-blind, placebo-controlled REGAIN study (ClinicalTrials.gov identifier: NCT01997229), producing clinically meaningful improvements in activities of daily living (ADL), muscle strength, functional ability and quality of life (QOL) in patients with anti-acetylcholine receptor antibody-positive (AChR^+^) refractory gMG.^2^ In clinical practice, there is no consensus regarding which kind of patients with gMG are selected to preferentially receive eculizumab.

The purpose of the present study was to determine the suitable indications for treatment with eculizumab for refractory gMG in Japan.

## Patients and methods

**Figure 1.**
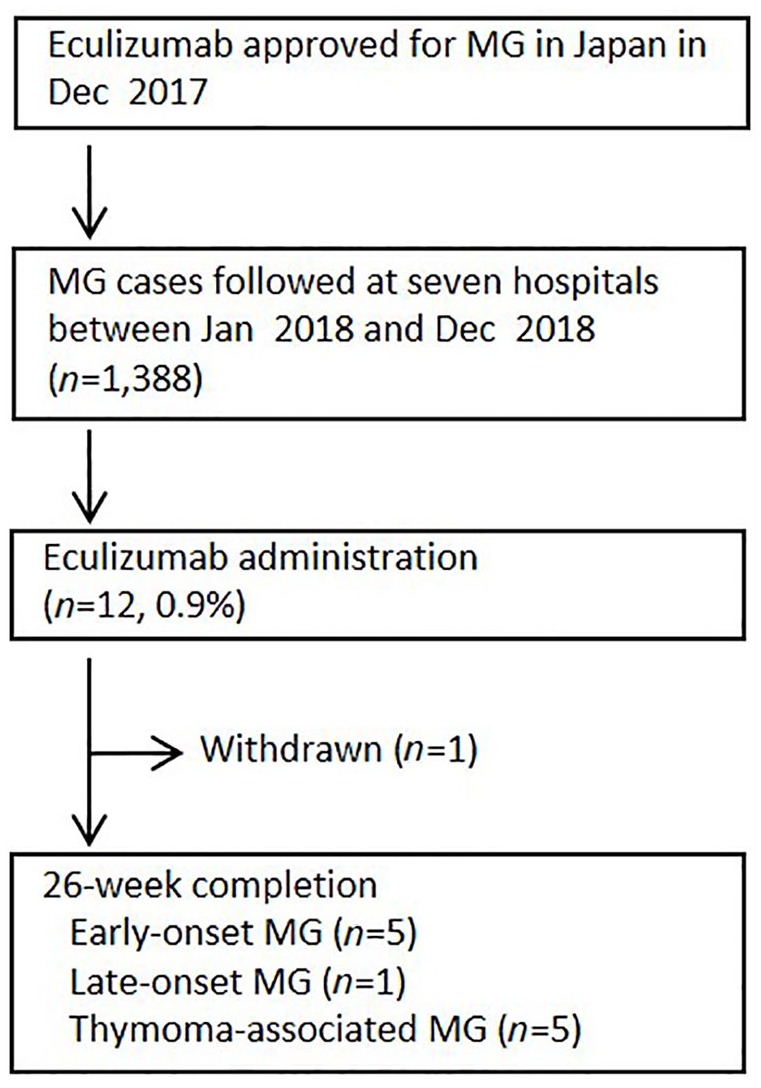
Study flow. MG, myasthenia gravis.

Eculizumab was approved by the Japanese Government for the treatment of AChR^+^ refractory gMG in December 2017 (Figure 1). Between January and December 2018, we followed 1388 patients with MG who were treated at seven hospitals located in Tokyo and Chiba. These hospitals included Keio University, Tokyo Medical University, Tokyo Women’s Medical University, Juntendo University, Chiba University, Neurology Clinic Chiba, and International University of Health and Welfare. The diagnosis of MG was based on clinical findings (fluctuating symptoms with easy fatigability and recovery after rest) along with the amelioration of symptoms after an intravenous administration of acetylcholinesterase inhibitors, decremental muscle response to a train of low-frequency repetitive nerve stimuli, or the presence of autoantibodies.^1^ Clinical status and severity were determined by the recommendations of the Myasthenia Gravis Foundation of America (MGFA).^6^

The schedule for eculizumab involved a dose of 900 mg on day 1 and weeks 1, 2, and 3, and 1200 mg every second week thereafter. All patients received *Neisseria meningitidis* vaccination according to local guidelines at least 2 weeks before starting eculizumab. Postmarket surveillance of all patients treated with eculizumab was required by the Japanese Pharmaceuticals and Medical Devices Agency. All clinical information was collected after the patients provided written informed consent. All study protocols were specifically approved by the institutional review board of the ethics committee of each institution (Keio University IRB 20090278). These clinical investigations were conducted according to the principles expressed in the Declaration of Helsinki.

Changes in clinical scores from baseline at week 26 were analyzed using the Mann–Whitney *U*-test. Values of *p* < 0.05 were considered statistically significant. Statistical analyses were performed using JMP version 9 statistical software (SAS Institute Inc., Cary, NC, USA).

## Results

### Baseline characteristics

Among 1388 patients with MG treated at seven hospitals, 12 (0.9%) patients received treatment with eculizumab. A total of 11 patients completed 26 weeks of treatment with eculizumab (Table 1). All of the patients that received eculizumab were injected for the first time except one (patient 5), who was previously included in the REGAIN study. He did not join the extension of the REGAIN study and experienced a worsening of MG thereafter. All 11 patients had AChR^+^ refractory gMG at a mean age of 43 years (22–67 years). They included eight women and three men. The disease subtypes represented included five cases of early onset MG, one of late-onset MG, and five of thymoma-associated MG. All patients suffered from severe gMG with an MGFA classification of III or higher. In fact, the mean quantitative MG (QMG) score and the MG-ADL score were 18.6 and 10.8, respectively, at the start of the eculizumab treatment.

**Table 1.** Clinical features of 11 patients with refractory generalized myasthenia gravis.

Patient/age/sex	Onset age	Type	MGFA class	QMG score	MG-ADL score	Anti-AChR (nM)	Treatment (daily dose)	Rescue treatment during previous 1 year
1/22/F	21	EOMG	V	35	17	80	PSL 20 mg	PE 2 times every 3 weeks
2/23/F	17	EOMG	V	20	10	12	PSL 15 mg, tacrolimus 3 mg	IVIg 3 course, IA 2 courses
3/33/F	12	EOMG	V	17	9	29	PSL 15 mg, tacrolimus 3 mg	IVIg 2 courses
4/33/F	31	EOMG	IIIa	19	17	0.8	PSL 7 mg/d, cyclosporin 250 mg	IVIg 3 course, IA 1 course
5/36/M	29	EOMG	IIIa	17	13	280	PSL 5 mg, tacrolimus 3 mg	IA 1 course
6/40/F	19	TMG	V	18	5	120	PSL 20 mg, tacrolimus 3 mg	IVIg 1 course, PE 2 course, DFPP 2 courses, IA 1 course
7/53/F	46	TMG	V	31	15	44	PSL 15 mg (AD), tacrolimus 2 mg	IVIg 1 course, IA 1 course
8/55/M	49	TMG	V	12	6	26	PSL 11 mg (AD), tacrolimus 3 mg	IVIg 1 course, IA 1 course, intravenous steroid pulse every 2 weeks
9/55/M	50	LOMG	IIIa	9	10	14	PSL 15 mg, tacrolimus 3 mg	IVIg 3 course
10/61/F	49	TMG	IVb	16	12	21	PSL 8 mg, tacrolimus 3 mg	IVIg 3 course, IA 6 courses
11/67/F	66	TMG	V	11	5	23	PSL 20 mg, tacrolimus 3 mg	IVIg 1 course, IA 5 courses, DFPP 1 course

AChR, acetylcholine receptor; AD, alternative day; DFPP, double-membrane filtration plasmapheresis; EOMG, early onset MG; F, female; IA, immunoadsorption using a tryptophan column; IVIg, intravenous immunoglobulin; LOMG, late-onset MG; M, male; MG, myasthenia gravis; MG-ADL, MG activities of daily living; MGFA, MG Foundation of America; PE, plasma exchange; PSL, prednisolone; QMG, quantitative MG; TMG, thymoma-associated MG.

All 11 patients received corticosteroids and additional ISTs. As calcineurin inhibitors are approved by the Japanese Government, tacrolimus and cyclosporin were used in nine patients and one patient, respectively. Extended thymectomy was performed in eight patients. Notably, all patients required rescue therapy. Frequent rescue therapy consisting of IVIg, plasmapheresis, and intravenous steroid pulse was performed three times or more during the previous year in nine patients. Plasmapheresis included plasma-exchange, double-membrane filtration plasmapheresis, and immunoadsorption, using a tryptophan column. Overall, three patients had MG-ADL scores of six points or fewer, because they underwent rescue therapy and showed clinical improvement just before the start of eculizumab. However, these patients repeatedly experienced exacerbation of MG soon after the rescue treatment.

Patient 6 had her first baby by vaginal delivery at the age of 38 years with no neonatal MG. Although her MG was stable during pregnancy, she experienced MG worsening at 4 months after the delivery. She received daily 20 mg of prednisolone and two cycles of IVIg, however, clinical improvement was not remarkable. Eculizumab was added at 6 months after the delivery.

### Case presentation

**Figure 2.**
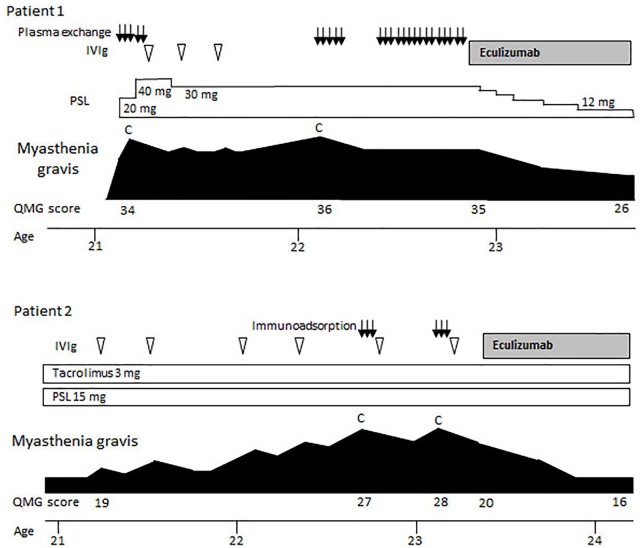
Clinical courses of patient 1 and patient 2. C, myasthenic crisis; IVIg, intravenous immunoglobulin; PSL, prednisolone (daily dose), QMG; quantitative myasthenia gravis.

Patient 1. A 21-year-old female with weakness of the neck and limb muscles and dyspnea was admitted to our hospital (Figure 2). Her symptoms rapidly progressed to myasthenic crisis and she was treated with plasma exchange. In addition, she had adverse reactions to drugs including hepatotoxicity induced by calcineurin inhibitors and leukocytopenia induced by IVIg. Thus, her treatment choice was limited to prednisolone and plasma exchange. After an internal shunt was created in her right forearm, she underwent two sessions of plasma exchange every 3 weeks to avoid myasthenic crisis. Eculizumab treatment was started with add on 30 mg/day of prednisolone. During the following 6 months, her symptoms gradually improved. Her QMG score was 26, which was greatly improved from her worst score of 36. She has residual weakness, however, she does not use a wheelchair. She can walk with no assistance and gains employment at a movie company.

Patient 2. A 21-year-old female with a 4-year history of MG was referred to our hospital. A hyperplastic thymus was removed at the age of 17 years. She required oral prednisolone, tacrolimus and IVIg for the maintenance treatment. At the ages of 22 and 23 years, she experienced myasthenic crises and received immunoadsorption using a tryptophan column. Soon after being weaned from the mechanical ventilation, she started eculizumab treatment. During the follow-up of 12 months, she did not fall into a myasthenic crisis. Her QMG score was 16, which was greatly improved from her worst score of 28. As she has a nasal voice, other immunosuppressive treatment may be required; however, she works part-time at a convenience store and enjoys traveling with her friends.

### Follow-up observation

**Figure 3.**
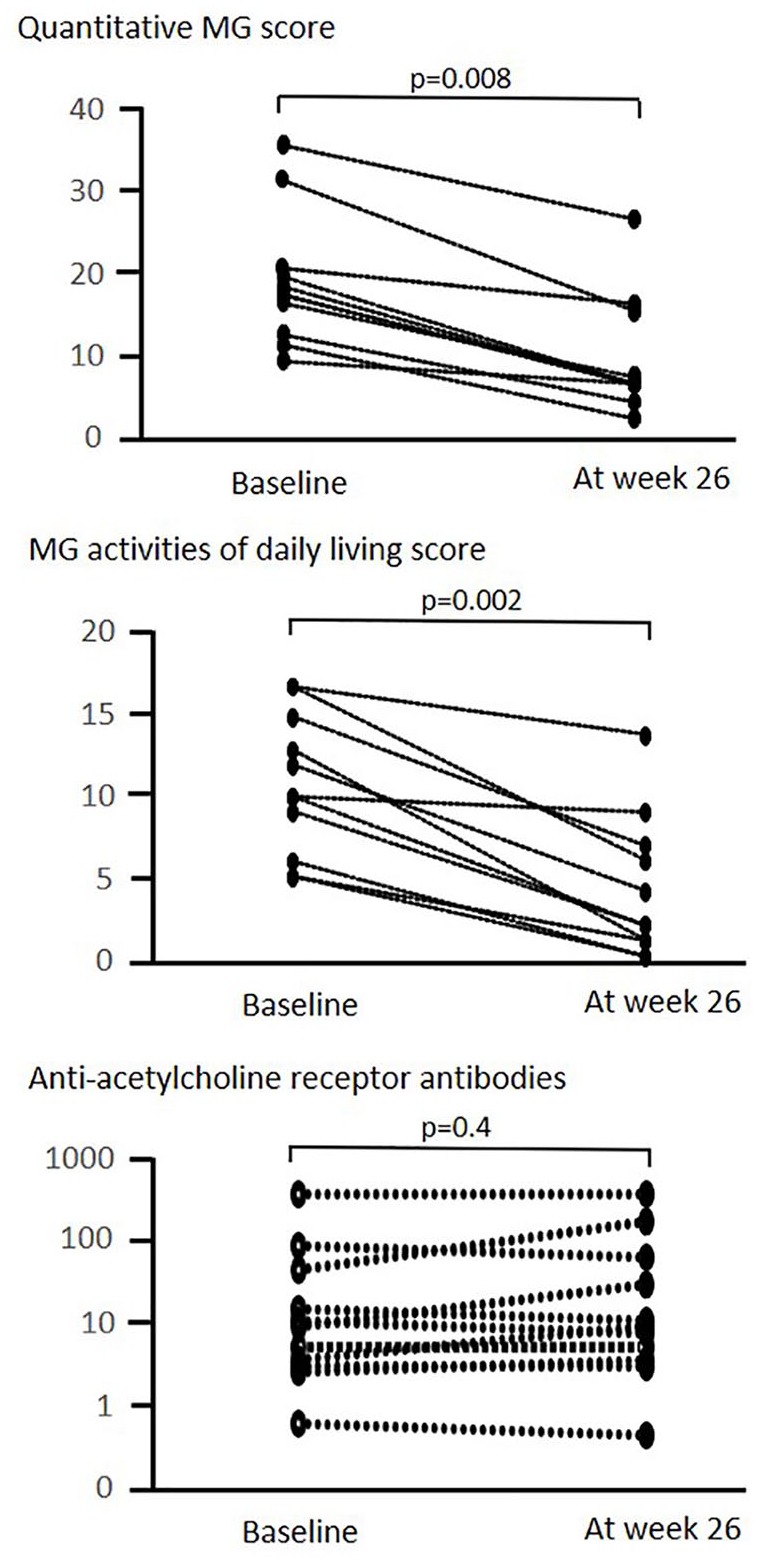
Changes in quantitative MG score, MG activities of daily living score and anti-acetylcholine receptor antibodies titers. MG, myasthenia gravis.

Table 2 indicates clinical features of 11 patients with gMG during follow-up periods. All 11 patients with refractory gMG showed rapid and marked improvement. The mean QMG score ranged from 18.6 at baseline to 9.1 at week 26 (*p* = 0.008; Figure 3). In addition, the mean MG-ADL score ranged from 10.8 at baseline to 4.2 at week 26 (*p* = 0.002). Postinterventional status revealed minimal manifestations, such as ptosis, diplopia, and limb weakness in seven patients and improvements in four patients, respectively. In accordance with these clinical scores, the QOL levels of all patients including the physiological, social, and psychological aspects of daily functioning were significantly improved. The QOL improvements of the 11 patients are indicated in Table 3.

**Table 2.** Clinical features during follow-up periods.

Patient	Follow-up period (months)	Thymus histology	Clinical symptoms	Times of myasthenic crisis	Postinterventional status
1	33	Not performed	Neck and limb weakness, dyspnea, ptosis	2	Improved
2	76	Hyperplasia	Nasal voice, dysphagia, dyspnea	3	Improved
3	254	Hyperplasia	Ptosis, diplopia, dyspnea	1	Minimal manifestations
4	33	Not performed	Dyspnea, leg weakness, dysarthria	0	Improved
5	86	Atrophy	Neck and limb weakness, diplopia, ptosis	0	Minimal manifestations
6	248	Thymoma (type B3, stage III)	Neck weakness, dysphagia, ptosis	3	Minimal manifestations
7	102	Thymoma (type B1, stage I)	Arm weakness, ptosis, diplopia	1	Improved
8	76	Thymoma (type B2, stage II)	Dyspnea, diplopia, ptosis	1	Minimal manifestations
9	67	Not performed	Dysarthria, ptosis, diplopia, limb weakness	0	Minimal manifestations
10	153	Thymoma (type AB, stage II)	Dysarthria, dysphagia, limb weakness	0	Minimal manifestations
11	19	Thymoma (type A, stage I)	Facial muscle weakness, dysphagia, nasal voice	1	Minimal manifestations

**Table 3.** Quality of life improvement in 11 patients with refractory generalized myasthenia gravis.

Patient	Most remarkable improvement in quality of life
1	No need for wheelchairs, employment at a movie company
2	Part-time work at a convenience store and travel with her friends
3	Disappearance of ptosis and use of mascara and eye liner
4	Returning to work as a teacher of ballet dancer
5	Performing night duty as a care attendant
6	Recovery from aggravation after delivery and caring for her child
7	Going out and washing her hair by her own efforts
8	Working as a licensed tax accountant
9	Achievement of the responsibility of being the president of a company
10	Handling all housework throughout the day
11	Eating any meal served to her and speaking fluently

We compared the QMG score between patients with MG with eculizumab treatment and those receiving at least two cycles of rescue treatment. Based on the QMG scores, treatment outcome was much better in the 11 patients with MG with eculizumab treatment than 20 refractory patients with MG who received rescue treatment alone (9.1 *versus* 16.2, *p* = 0.0009).

With regard to immunosuppressive therapy, seven patients were able to reduce their dose of prednisolone (mean 5.0 mg/day) at week 26. Reduction of the dose of tacrolimus was also achieved in two patients (from 4.5 mg to 3 mg and from 3 mg to 1 mg, respectively). It was noted that all but one patient did not require additional rescue therapy. Patient 4 received one course of plasma exchange 9 weeks after the start of eculizumab treatment because she had trouble breathing. The chronological changes of the anti-AChR antibodies titers did not show a constant tendency; the titers increased in four, decreased in three, and were unchanged in four patients (Figure 3).

### Adverse events

Common adverse events due to eculizumab include headache and nasopharyngitis. In the present study, two patients noticed mild headache, but they continued the eculizumab treatment. In contrast, a 36-year-old woman suffered from nausea and vertigo immediately after the first injection of eculizumab. A neurological examination revealed no abnormality. Head magnetic resonance imaging and otolaryngeal examination showed no significant findings. After withdrawal of the eculizumab, she received IVIg every 2 months to prevent myasthenic crisis.

## Discussion

We administered eculizumab to 12 patients who were AChR^+^ with gMG over the course of 1 year, soon after its approval in Japan. They represented only 0.9% of the total patients with MG treated at seven referring hospitals, suggesting that they were the patients with the most suitable indications for eculizumab for gMG. All but one completed 26 weeks of the eculizumab treatment. The mean reduction in QMG score from baseline at week 26 was 9.5 in the present study; this reduction was 4.6 in the REGAIN study.^2^ The mean reduction in MG-ADL score from baseline at week 26 was 6.6 in the present study; it was 4.2 in the REGAIN study. In addition, all 11 patients with gMG were greatly satisfied with their improvements in QOL. We demonstrate that eculizumab provided remarkable benefits for refractory gMG in practical real-world experience as well as in the REGAIN study. Recently, Muppidi and colleagues reported the long-term safety and efficacy of eculizumab in gMG based on a median duration of almost 2 years of eculizumab treatment using the REGAIN extension study.^7^

We emphasize that there is a marked discrepancy between the entry criteria for the REGAIN study and real-world indications for treatment.^2^ The inclusion criteria for the REGAIN study were as follows: (a) aged ⩾18 years; (b) AChR^+^ gMG; (c) MG-ADL score of 6 or higher; (d) failed treatment with two or more ISTs or at least one IST with requirement for chronic IVIg or plasma-exchange therapy over the preceding 12 months; and (e) MGFA class II, III, or IV. On the other hand, patients with gMG suffering myasthenic crisis (MGFA class V) at screening, or who required treatment with IVIg or plasma exchange within the 4 weeks before randomization were excluded. Patients were also excluded if they had a history of thymoma or other thymic neoplasm or had undergone thymectomy in the 12 months before the screening. Based on our experience and previous reports, we think that eculizumab may be useful in gMG patients under the following conditions.^8,9^

First, we consider myasthenic crisis to be the best indication for eculizumab. In the present study, 7 of 11 patients had experienced myasthenic crisis. Overall, two patients in particular, as presented in Figure 2, remained free from myasthenic crisis while under treatment with eculizumab without additional IVIg nor plasmapheresis. During the extension study of eculizumab with a median duration of 22.7 months, only 3 (2.6%) of 117 patients deteriorated to myasthenic crisis.^1^ We consider that eculizumab should be preferentially selected for patients with MG who have undergone repeated myasthenic crises. Eculizumab is also expected to lead to rapid improvement from myasthenic crises and to enable patients to be weaned from mechanical ventilation. Yeo and Pleitez described a 79-year-old woman with myasthenic crisis who was successfully treated with eculizumab, which had been an effective treatment for her previous disease of atypical hemolytic uremic syndrome.^8^

Second, we think that thymoma-associated MG is also an appropriate indication for eculizumab based on the present study. The clinical features of thymoma-associated MG differ from those of nonthymomatous MG, including a more severe involvement of MG, the development of other autoimmune disorders, and MG relapse accompanied by thymoma recurrence.^10–12^ Thymoma should be considered a potentially malignant tumor requiring prolonged follow-up for both thymoma recurrence and MG relapse. In this regard, Amano and colleagues reported a 48-year-old man with thymoma-associated gMG presenting with severe muscle weakness.^9^ The motor symptoms improved remarkably with the administration of eculizumab, as did taste disorder, which was one of the nonmotor symptoms associated with thymoma-associated MG.

Third, eculizumab is also expected to be well tolerated for patients with MG during pregnancy, although there are no reports on successful pregnancies in female patients with MG who have used eculizumab. It is known that pregnancy does not affect MG in any consistent way, with no increased risk of severe deterioration or myasthenic crisis.^13^ However, during the first weeks and months postpartum, the risk of symptoms worsening is moderately increased, mainly because of stress and new demands. Kelly and colleagues reported the safety and efficacy of eculizumab in 61 pregnant women with paroxysmal nocturnal hemoglobinuria by examining the birth and developmental records of the children.^14^ They concluded that eculizumab provided a benefit to women during pregnancy, as evidenced by a high rate of fetal survival and a low rate of maternal complications.

We have to consider the medication costs, as noted by Edmundson and Guidon.^15^ Eculizumab is the most expensive treatment for MG in Japan (60,000,000 Yen per year) as well as in the United States (US$720,000 per year). In Japan, the medical costs of MG treatment are paid at public expense based on the Government’s programs for treating specified intractable disease. If eculizumab was to be widely used in patients with gMG, the financial resources used to pay the medical costs would probably collapse. To maintain the medical security of approximately 30,000 patients with MG in the future, Japanese neuromuscular experts should keep in mind the appropriate indications for eculizumab for refractory gMG.

One limitation of this study is that this was an observational study with a limited number of patients. In addition, our postmarketing surveillance is inherently prone to bias, particularly in patient selection. A prospective study with a large number of patients and longer follow-up period is necessary to draw a definite conclusion. In addition, a single missense C5 heterozygous mutation (c.2654G>A) was identified as a poor responder to eculizumab in patients with paroxysmal nocturnal hemoglobinuria.^16^ We did not evaluate the genetic variation in C5 and did not measure CH50 nor antistriational antibodies in all patients, although eculizumab was effective for all our patients. We are now conducting a prospective multicenter study on the pathophysiological features of patients with gMG treated with eculizumab in Japan (PREDICT-MG). Biomarkers for predicting a good response to eculizumab will be determined by examining chronological changes in the blood complement proteins, serum MG-related antibodies, and various inflammatory markers.

**Figure 4.**
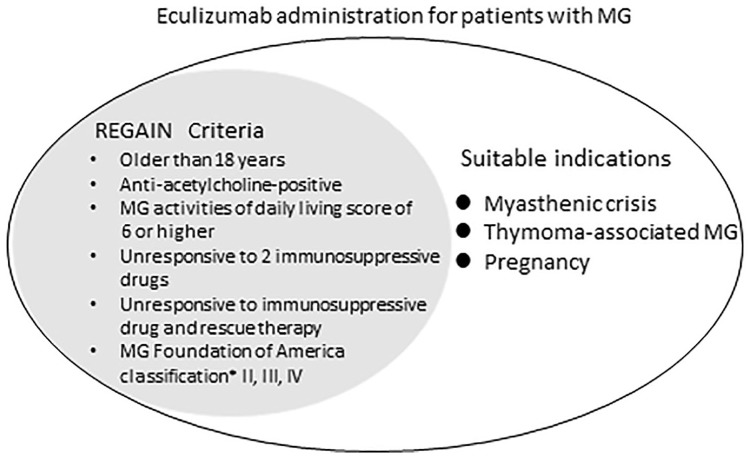
Suitable indications of eculizumab for MG. MG, myasthenia gravis. ^*^MG Foundation of America classification was graded at the screening of the REGAIN study.

In conclusion, we consider that patients with refractory gMG with myasthenic crisis, thymoma-associated MG, and pregnancy may be suitable recipients for eculizumab administration (Figure 4).
